# The inhibition of high ammonia to *in vitro* rumen fermentation is pH dependent

**DOI:** 10.3389/fvets.2023.1163021

**Published:** 2023-03-30

**Authors:** Junshi Shen, Wenjin Zheng, Yixuan Xu, Zhongtang Yu

**Affiliations:** ^1^Laboratory of Gastrointestinal Microbiology, National Center for International Research on Animal Gut Nutrition, Nanjing Agricultural University, Nanjing, China; ^2^Ruminant Nutrition and Feed Engineering Technology Research Center, College of Animal Science and Technology, Nanjing Agricultural University, Nanjing, China; ^3^Department of Animal Sciences, The Ohio State University, Columbus, OH, United States

**Keywords:** ammonium chloride, free ammonia, microbiota, pH, rumen fermentation, urea

## Abstract

Ammonia is an important rumen internal environment indicator. In livestock production, feeding a large amount of non-protein nitrogen to ruminants will create high ammonia stress to the animals, which increases the risk of ammonia toxicity. However, the effects of ammonia toxicity on rumen microbiota and fermentation are still unknown. In this study, an *in vitro* rumen fermentation technique was used to investigate the effects of different concentrations of ammonia on rumen microbiota and fermentation. To achieve the four final total ammonia nitrogen (TAN) concentrations of 0, 8, 32, and 128 mmol/L, ammonium chloride (NH_4_Cl) was added at 0, 42.8, 171.2, and 686.8 mg/100 mL, and urea was added at 0, 24, 96, and 384 mg/100 mL. Urea hydrolysis increased, while NH_4_Cl dissociation slightly reduced the pH. At similar concentrations of TAN, the increased pH of the rumen culture by urea addition resulted in a much higher free ammonia nitrogen (FAN) concentration compared to NH_4_Cl addition. Pearson correlation analysis revealed a strong negative correlation between FAN and microbial populations (total bacteria, protozoa, fungi, and methanogens) and *in vitro* rumen fermentation profiles (gas production, dry matter digestibility, total volatile fatty acid, acetate, propionate, etc.), and a much weaker correlation between TAN and the above indicators. Additionally, bacterial community structure changed differently in response to TAN concentrations. High TAN increased Gram-positive Firmicutes and Actinobacteria but reduced Gram-negative Fibrobacteres and Spirochaetes. The current study demonstrated that the inhibition of *in vitro* rumen fermentation by high ammonia was pH-dependent and was associated with variations of rumen microbial populations and communities.

## Introduction

Ruminants, which provide almost all of the milk and much of the meat consumed by humans globally, are of great importance in agricultural production (1, 2). The rumen is a unique digestive and metabolic organ of ruminants, and it contains a diverse microbiota consisting of bacteria, protozoa, fungi, archaea, and viruses (3). The rumen microbes can produce enzymes to digest crude fibers that cannot be digested by the host itself to short-chain fatty acids (primarily acetate, propionate, and butyrate), providing the main energy source and fat synthesis precursors for the host (4). Besides, these microbes also synthesize microbial proteins from ammonia, which is derived from deamination of amino acids and hydrolysis of non-protein nitrogen (NPN) such as urea, providing the primary protein synthesis precursors for the host (5, 6). A proper rumen internal environment (pH, ammonia concentration, etc.) is critical to ensure efficient degradation of crude fibers and microbial protein synthesis in the rumen (3).

Ammonia can be utilized by many rumen microbes to synthesize microbial protein. In order to lower production costs, NPN (primarily urea) is used to replace part of high-quality protein sources (such as soybean meal) fed to ruminants (6–8). However, if a high dose of NPN is added, rumen ammonia concentration can increase rapidly, leading to ammonia toxicity (8). Total ammonia nitrogen (TAN) in aqueous phase exists in two different molecular forms, NH_3_ as free ammonia nitrogen (FAN) or NH${}_{4}^{+}$ as ammonium ions. The equilibrium concentration between NH_3_ and NH${}_{4}^{+}$ follows the Henderson-Hasselbalch equation and depends on pH and temperature (9). One previous study has found that ammonia toxicity is positively correlated with rumen pH and blood ammonia, but not with rumen TAN (10). Generally, the rumen temperature is relatively stable. Therefore, rapid increase of FAN concentration in the rumen at high pH is probably the primary cause for the rapid increase of ammonia absorption through the rumen epithelium leading to ammonia toxicity (7, 8, 11).

High ammonia stress is a major problem frequently encountered in anaerobic digestion for biogas production, a technology commonly used to treat organic wastes (9). The performance of an anaerobic reactor is directly associated with the structure of the microbial community present therein (12). Free ammonia levels are considered the foremost cause of inhibition of methanogens due to its high permeability to cell membrane (9, 13). For anaerobic digestion in biogas production, the goal is to maximize methane yield while reducing volatile fatty acid (VFA) accumulation (14). In contrast, for rumen fermentation, the goal is to maximize feed digestion and VFA production while reducing methane emissions (15). Besides, the operation parameters of anaerobic digesters (microbial composition, pH, temperature, ammonia concentration, etc.) are considerably different from those of rumen fermentation. Therefore, the specific response mechanism of the microbiota to ammonia toxicity may be different between the two different anaerobic digestion systems. Previous studies in ruminants have focused on the effects of ammonia toxicity on animal health (10, 16, 17). To our knowledge, however, no studies had reported the effects of ammonia toxicity on rumen microbiota even though ammonia toxicity adversely affects the animals. It is hypothesized that high FAN may affect the structure of rumen microbiota, leading to the inhibition of rumen fermentation.

Different NPN has varied acidity or alkalinity. Free NH_3_ produced from urea hydrolysis mediated by microbial urease in the rumen is a weak base, and it can neutralize the acidity produced by rumen fermentation and buffer the rumen pH to some extent (7). In contrast, NH_4_Cl, which is also an NPN additive commonly used in ruminants, is a weak acid after it is dissolved in water (17, 18). In this study, a rumen pH and ammonia level model was implemented by changing the amount and type of NPN (NH_4_Cl vs. urea) in an *in vitro* rumen fermentation system, and this model was used to investigate the effects of NH_4_Cl and urea addition on rumen microbial composition and fermentation profiles. The results helped reveal the microbial mechanism by which high ammonia inhibit rumen fermentation and could inform the improvement of ammonia utilization by the rumen microbiota.

## Materials and methods

### Experimental design

The experiment was designed in a 2 × 4 factorial arrangement: two nitrogen sources (NH_4_Cl and urea) and four TAN levels (0, 8, 32, and 128 mmol/L). The rumen temperature is maintained quite stable varying within the narrow range of 38–41°C, and rumen TAN concentration and pH fluctuated between 1–40 mM and 5.5–7.2, respectively (19). In the present study, the tested TAN levels covered the TAN concentrations found in the rumen. To achieve the four final TAN concentrations of 0, 8, 32, and 128 mmol/L, NH_4_Cl was added at 0, 42.8, 171.2, and 686.8 mg/100 mL (A-0, A-8, A-32, and A-128), and urea was added at 0, 24, 96, and 384 mg/100 mL (U-0, U-8, U-32, and U-128). Each treatment had four replicates.

### Ruminal inoculum and *in vitro* incubation

All animal protocols were approved by the Animal Care and Use Committee of Nanjing Agricultural University (protocol number: SYXK2017-0007).

Three rumen cannulated male sheep (Body weight = 32 ± 2 kg) served as ruminal fluid donors for this *in vitro* study. The diet fed to these sheep contained (% DM basis) 45% forage (25% corn silage and 20% peanut vine) and 55% concentrate (42% ground corn, 4% soybean meal, 4% wheat bran, and 5% premix). The dietary nutrient composition (DM basis) of crude protein (CP), neutral detergent fiber (NDF), acid detergent fiber (ADF), and ether extract (EE) were 15.6, 32.7, 20.4, and 3.1% respectively, and digestible energy (DE) was 13.8 MJ/kg, which met the feeding standards of meat-producing sheep and goats (20). The sheep were fed twice daily at 08:00 and 18:00, and they had free access to feed and water. Ruminal contents were collected through rumen cannula from the three donor sheep before morning feeding, mixed with an equal volume, and then poured into a sterilized bottle (1,000 mL) leaving no headspace in the bottle, which was taken to the laboratory within 30 min. The mixed rumen sample was then squeezed through four layers of cheesecloth into a flask under a continuous flux of CO_2_ in a water bath kept at 39°C until use.

The *in vitro* batch fermentation was performed in 180 mL serum bottles. The fermentation substrate was the same feed fed to the three sheep that donated the rumen sample. The buffered medium for the *in vitro* fermentation was prepared anaerobically as described by Theodorou et al. (21) with minor modification: Ammonium bicarbonate was replaced with equivalent amounts of sodium bicarbonate to eliminate the background nitrogen content in the buffered medium. The anaerobic buffer medium and strained rumen fluid inoculum were combined in each bottle in a 9:1 (v/v) ratio under anaerobic conditions. A 100-mL mixture was immediately dispensed into each incubation bottle containing 1 g of ground feed substrate and respective additions of NH_4_Cl and urea. To prevent exposure to air, the headspace of the bottles was continuously flushed with CO_2_ before they were each sealed with a butyl rubber stopper and secured with an aluminum crimp seal. The *in vitro* cultures were incubated at 39°C for 24 h in a water bath with intermittent shaking by hand after gas measurement at each designed time point.

### Sampling and chemical analysis

Gas production was measured at 0.5, 2, 4, 6, 8, 12, and 24 h using a pressure transducer (21). After gas measurement at each time point, 1 mL of each culture was collected for subsequent analysis for ammonia-N using a colorimetric method (22). At the end of the 24 h of incubation, the pH value of each *in vitro* culture was measured immediately using a portable pH meter. Then, 1 mL of culture each was preserved by adding 0.2 mL of 25% HPO_3_ for VFA analysis using gas chromatography (7890A, Agilent, UK) according to the method described by Mao et al. (23). Also, 1 mL of culture each was collected for DNA extraction and subsequent microbial analysis. All the samples were stored at −20°C until analyses. The remaining content of each culture was filtered through a filter bag (ANKOM Technology, USA) to analyze apparent dry matter (DM) digestibility gravimetrically (24).

The free ammonia concentrations at 24 h after incubation were calculated based on the following equation as described by Rajagopal et al. (9):


$$\begin{array}{l} \text{FAN}=\text{TAN}\times \;{\left(1+\;\frac{10^{-pH}}{10^{-\left(0.09018+\frac{2729.92}{T\left(K\right)}\right)}}\right)}^{-1} \end{array}$$

where FAN is the concentration of free ammonia (mmol/L); TAN is the concentration of total ammonia (mmol/L); T(K) is the rumen temperature (Klevin), which was fixed at 312.15 K (39°C) in the current study; and pH is the pH value of each culture measured after 24 h incubation.

### DNA extraction and real-time quantitative PCR analysis

Microbial genomic DNA of the rumen culture samples was extracted using the bead-beating and phenol-chloroform extraction method (25). The DNA integrity was examined using agarose (1.2%) gel electrophoresis, and the DNA quantity of each sample was determined using a Nanodrop 2000 (Thermo Fisher Scientific, Inc., Madison, USA).

The PCR primers used for real-time quantitative PCR (qPCR) of total bacteria (25), fungi (26), protozoa (27), and methanogens (28) are listed in Supplementary Table 1. Real-time qPCR was performed on a StepOnePlus system (Applied Biosystems, California, USA) using the SYBR Premix Ex Taq dye (Takara Bio Inc.). Copies of 16S rRNA gene (total bacteria), methyl coenzyme-M reductase alpha-subunit gene (*mcr*A, methanogens), and 18S rRNA gene (fungi and protozoa) in each sample were performed in triplicate. Standard curves were generated using 10-fold serial dilutions of purified plasmid DNA containing the target gene sequences of each microbial group. The absolute abundance of each microbial population was expressed as copies of the target gene/mL of each sample.

### Illumina sequencing of 16S rRNA gene amplicons and data analysis

The V3-V4 region of the 16S rRNA gene was amplified using primers 341F (5′-CCTACGGGAGGCAGCAG-3′) and 806R (5′-GGACTACHVGGGTWTCTAAT-3′). Unique barcodes were added to the 5'end of both primers for multiplexing. PCR products were verified on agarose gel (2%, w/v), and the expected bands were each extracted and purified using the QIAquick PCR Purification Kit (Qiagen, CA, USA). The concentrations of the purified DNA amplicons were each quantified using a QuantiFluor^®^ dsDNA kit (Promega, Madison, WI, USA). Amplicons from all the samples were mixed in equal ratio and sequenced on an Illumina MiSeq platform to produce 250-bp paired-end reads. The raw sequence reads were deposited into the NCBI Sequence Read Archive (SRA) database under the accession number PRJNA940661.

Raw FASTQ files were de-multiplexed, quality-filtered (minimum Q score = 25), and analyzed using QIIME 1.9.1 (29). Operational taxonomic units (OTUs) were *de novo* clustered using UPARSE with a 97% sequence similarity (30), and possible chimeras were identified and removed using UCHIME (31). The most abundant sequence within each OTU was selected as the representative sequence and was taxonomically classified based on the SILVA database (version 138) (32). Sequences identified as of chloroplasts or mitochondria were removed before further analysis. The representative sequence of each OTU for each sample was aligned using MUSCLE (33), and the alignment was used to create a phylogenetic tree using FASTTREE (34). Principal coordinates analysis (PCoA) was performed based on Bray-Curtis dissimilarity to reveal overall differences in the bacterial communities among the different treatments. Analysis of similarities (ANOSIM) was performed to determine group similarity, where 0 = indistinguishable and 1 = dissimilar (35).

### Statistical analysis

The real-time qPCR data were log-transformed to improve normality. Residual analysis was used to determine if transformation of variables was needed. If needed, cubic root transformations were performed. All data (*in vitro* rumen fermentation parameters, microbial populations quantified by qPCR, relative abundances of bacteria at the phylum and genus levels) were analyzed using the MIXED procedure of SAS version 9.4 (SAS Institute Inc., Cary, NC) in a 2 (nitrogen source) × 4 (ammonia level) factorial design. The model included nitrogen source, ammonia level, and interaction of nitrogen source × ammonia level as fixed effects. Degrees of freedom were calculated using the Kenward-Roger option. Mean separation was performed using the Tukey multiple range test. Differences were considered statistically significant at *P* ≤ 0.05. Pearson correlation coefficients were calculated using SAS version 9.4 to examine the correlation between TAN or FAN and *in vitro* rumen fermentation parameters, microbial populations, relative abundance of rumen bacteria at the genus level (data of A0 and U0 were not included for correlation analysis because of the insufficient ammonia concentration). A significant correlation was declared at *P* ≤ 0.05.

## Results

### Rumen total ammonia, pH, and free ammonia concentration

**Figure 1:**
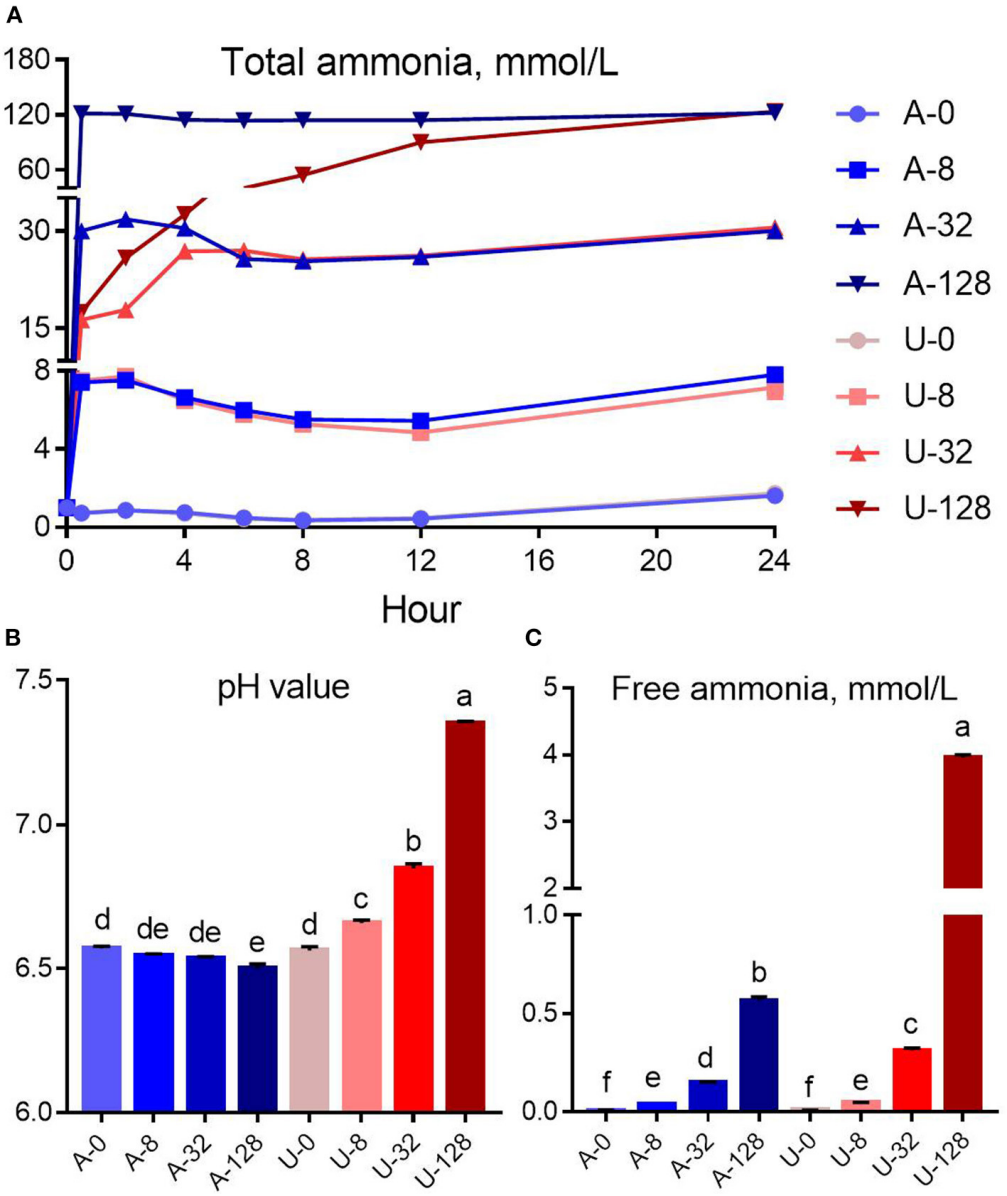
Effects of NH_4_Cl and urea addition on the dynamic change of total ammonia **(A)**, pH value **(B)**, and free ammonia concentration **(C)** at 24 h of *in vitro* incubation. There was significant interaction (*P* < 0.01) between nitrogen source and ammonia level with respect to pH value and free ammonia concentration. Different letters on the top of the bars in each figure panel denote significant differences (*P* < 0.05).

The addition of the pre-set amounts of urea and NH_4_Cl resulted in final TAN levels similar to that of the design, but at different rates (Figure 1A). NH_4_Cl dissociation can reach the target TAN level instantly after inoculation, while urea hydrolysis, which is catalyzed by urease, needs time to release ammonia. In the U-8 group, urea was hydrolyzed completely within 0.5 h, but it took about 4 and 12 h to complete hydrolysis of urea in the U-32 and U-128 groups, respectively.

There was significant interaction (*P* < 0.01) between nitrogen source and ammonia level for pH value and FAN concentration. The addition of urea and NH_4_Cl had different effects on *in vitro* rumen pH (Figure 1B). With the increase of urea addition, the rumen pH increased from 6.56 to 7.35. In contrast, NH_4_Cl addition slightly reduced the pH of the *in vitro* rumen culture. But the reduction magnitude is not very small, < 0.07 pH units (from 6.57 to 6.50).

In the present study, the increased *in vitro* rumen pH in response to the urea addition resulted in a much higher FAN concentration (*P* < 0.05) compared to NH_4_Cl at a similar TAN level of 32 or 128 mmol/L (Figure 1C). For instance, the pH of the U-32 group (6.84) was 0.30 unit higher than that of the A-32 group (6.54), while the FAN of the U-32 group (0.31 mmol/L) was more than twice of that of the A-32 group (0.15 mmol/L). In contrast, the pH of the U-128 group (7.35) was 0.85 unit higher than that of the A-128 group (6.50), but the FAN of the U-128 group (3.96 mmol/L) was 7 times higher than that of the A-128 group (0.57 mmol/L). Therefore, pH value is the key factor to determine the concentration of FAN in the rumen.

### Gas production, dry matter digestibility, and volatile fatty acids profile

**Figure 2:**
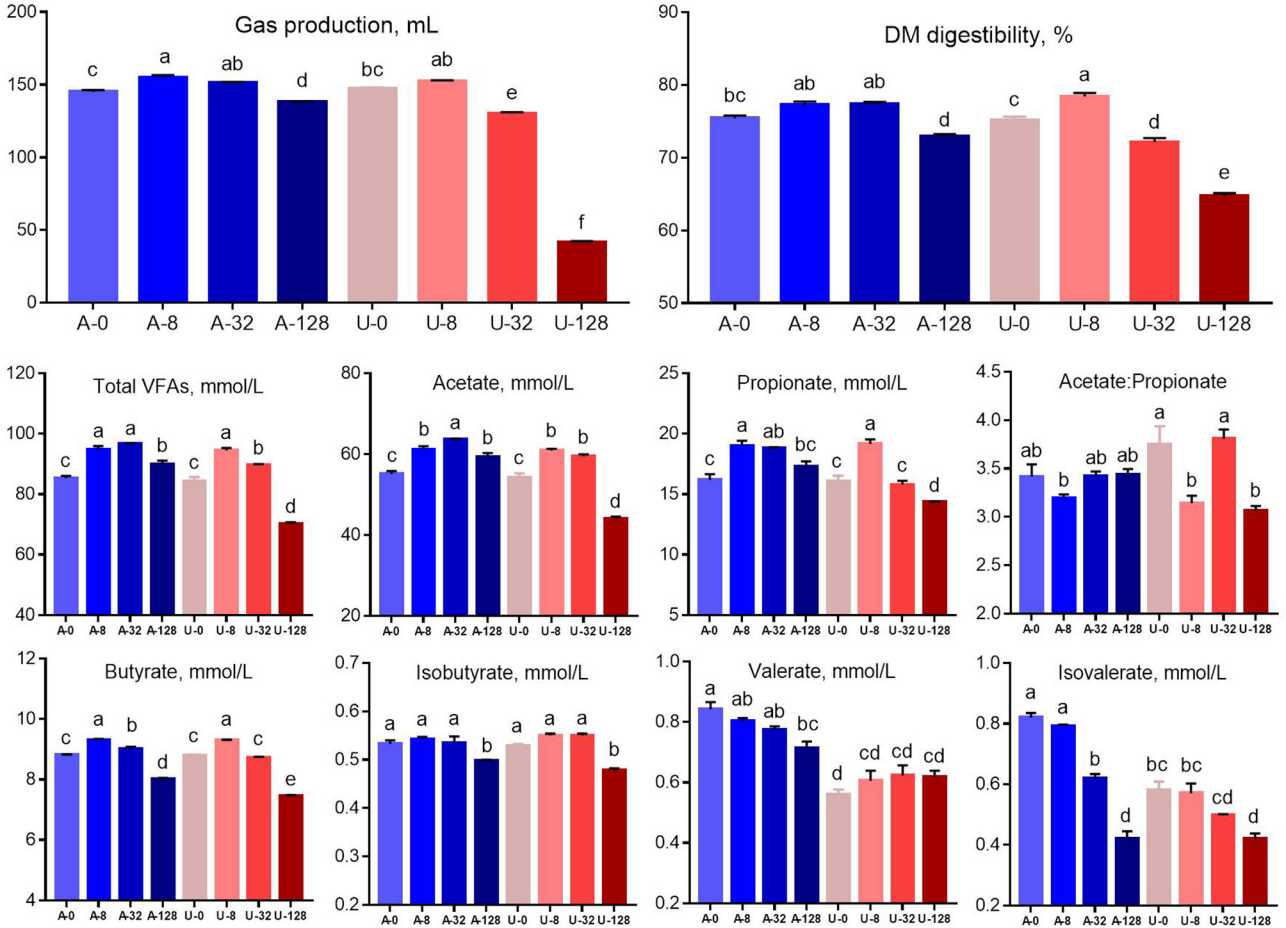
Effects of NH_4_Cl and urea addition on gas production, dry matter (DM) digestibility, and concentrations of volatile fatty acids. There was significant interaction (*P* < 0.01) between nitrogen source and ammonia level with respect to gas production, DM digestibility, and concentrations of volatile fatty acids. Different letters on the top of the bars in each figure panel denote significant differences (*P* < 0.05).

There was significant interaction (*P* < 0.01) between nitrogen source and ammonia level to gas production, DM digestibility, and concentrations of volatile fatty acids. Total gas production, DM digestibility, and concentration of total VFA, acetate, propionate, and butyrate increased when the TAN level was raised from 0 to 8 mmol/L (A-8 and U-8), but these characteristics showed different responses to the urea and NH_4_Cl additions higher than 8 mmol/L (Figure 2). When TAN reached 32 mmol/L by adding NH_4_Cl (A-32), gas production, DM digestibility, total VFA, and propionate concentration were similar to those observed in A-8 and U-8 (*P* > 0.05), but the above parameters was decreased significantly in A-128 group (*P* < 0.05). In contrast, the *in vitro* rumen fermentation was inhibited by U-32 (*P* < 0.05) and further inhibited by U-128, and the inhibition was much stronger than that observed in the A-128 group (*P* < 0.05). These results indicate that urea is more inhibitory to rumen feed digestibility and fermentation than NH_4_Cl at high concentrations within the current pH range (6.54–7.35).

### Microbial population

**Figure 3:**
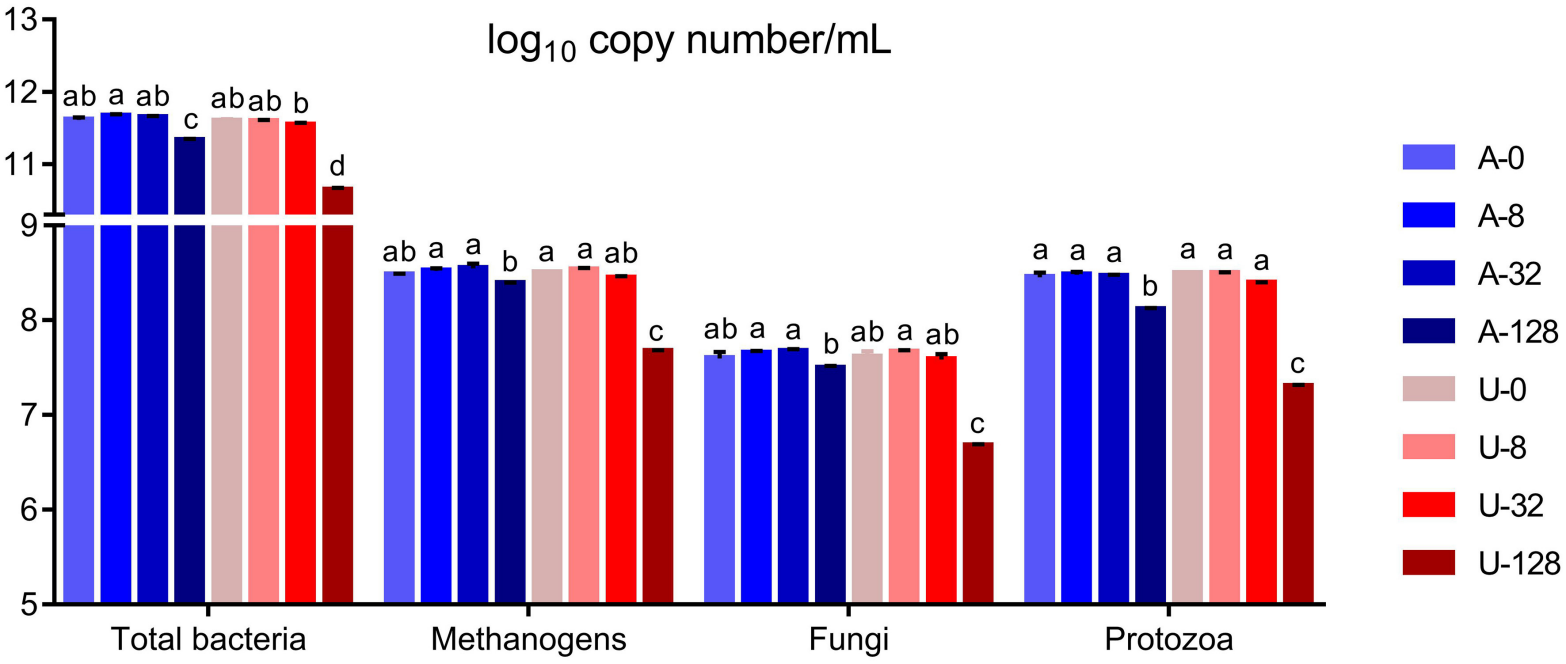
Effects of NH_4_Cl and urea addition on the absolute abundance of total bacteria, methanogens, protozoa, and fungi (log10 copy number of the target genes/mL) in the *in vitro* rumen mixed cultures. There was significant interaction (*P* < 0.01) between nitrogen source and ammonia level with respect to the abundance of total bacteria, methanogens, protozoa, and fungi. Different letters on the top of the bars in each figure panel denote significant differences (*P* < 0.05).

Significant interaction (*P* < 0.01) between nitrogen source and ammonia level was detected with respect to the absolute abundance of total bacteria, methanogens, protozoa, and fungi (Figure 3). In the present study, the abundance of total bacteria, fungi, and protozoa remained similar when TAN varied between 0 and 32 mmol/L (*P* > 0.05), but when TAN reached 128 mmol/L, the abundance of the above microbial groups decreased significantly (*P* < 0.05) irrespective of the ammonia-N source. However, U-128 had a greater inhibition than A-128 (*P* < 0.05). These results indicate that high TAN reduced the abundance of microbial populations, and increased FAN from urea hydrolysis might have aggravated the inhibitory effect.

### Bacterial community structure

**Figure 4:**
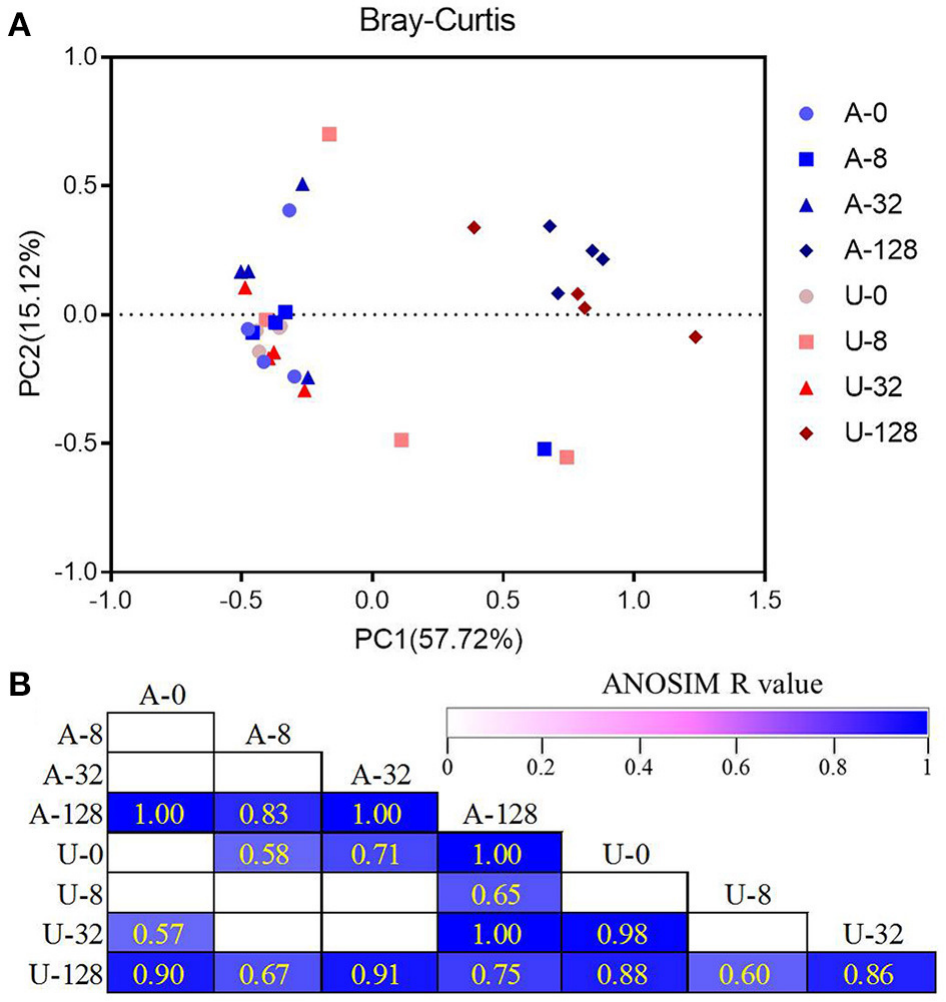
Principal coordinates analysis (PCoA) plots showing the overall differences in bacterial communities among the different treatments based on Bray-Curtis dissimilarity **(A)**. The statistical significance of the PCoA analysis of the overall bacterial communities among the treatments was analyzed using analysis of similarity (ANOSIM) **(B)**. ANOSIM *R*-values range from 0 (indistinguishable) to 1 (dissimilar), and ANOSIM *R*-values are showed only if *P* < 0.05, and cells are colored based on ANOSIM *R*-values.

Principal coordinates analysis based on Bray-Curtis dissimilarity showed clear separations of the microbiota between the highest TAN level of 128 mmol/L (A-128 and U-128) and other TAN levels (Figure 4A). Besides, the difference between A-128 and U-128, U-32 and A-0 or U-0, and A-32 and U-0 was also significant as analyzed using ANOSIM (*P* < 0.05, Figure 4B). These results indicate that the rumen bacterial community structure changed in response to the two different nitrogen sources and ammonia levels.

A total of 19 bacterial phyla were identified across all the treatments, with Bacteroidetes (32.12–47.82%), followed by Firmicutes (16.13–40.84%), Proteobacteria (11.17–26.53%), Fibrobacteres (0.03–9.00%), Tenericutes (1.77–7.10%), Spirochaetes (0.70–7.00%), Actinobacteria (0.91–8.68%), and Fusobacteria (0.04–2.42%) being the eight most predominant phyla, each of which was represented by more than 1.0% of the total sequences in at least one treatment (Table 1).

**Table 1:** Effects of NH_4_Cl and urea addition on the relative abundance of major ruminal bacterial phyla (each with a relative abundance ≥1.0% in at least one treatment).

**Item**	**NH**_**4**_ **Cl**				**Urea**				**SEM**	***P*** **-value**		
	**A-0**	**A-8**	**A-32**	**A-128**	**U-0**	**U-8**	**U-32**	**U-128**		**NS**	**AL**	NS*AL
Bacteroidetes	41.96	36.88	47.82	28.75	40.32	38.99	39.05	32.12	5.207	0.74	0.10	0.65
Firmicutes	20.33	20.69	16.13	40.84	21.87	20.18	21.47	39.06	3.402	0.64	< 0.01	0.74
Proteobacteria	11.92	26.53	15.94	13.99	11.17	22.21	18.26	14.57	3.286	0.81	< 0.01	0.78
Fibrobacteres	8.97	4.72	7.09	3.43	9.00	8.03	6.86	0.03	1.466	0.94	< 0.01	0.18
Tenericutes	6.91	3.35	1.83	1.77	7.10	4.41	4.06	5.31	0.841	< 0.01	< 0.01	0.24
Spirochaetes	7.00	4.80	8.39	0.70	6.92	4.36	6.34	0.66	1.163	0.43	< 0.01	0.80
Actinobacteria^1^	0.98^c^ (0.97)	1.06^c^ (1.21)	1.01^c^ (1.09)	2.04^a^ (8.68)	1.14^c^ (1.48)	0.95^c^ (0.91)	1.14^c^ (1.50)	1.65^b^ (4.63)	0.071	0.28	< 0.01	< 0.01
Fusobacteria^1^	0.33^d^ (0.04)	0.46^cd^ (0.10)	0.60^c^ (0.23)	0.99^b^ (1.01)	0.35^d^ (0.04)	0.47^cd^ (0.12)	0.62^c^ (0.25)	1.32^a^ (2.42)	0.051	0.01	< 0.01	0.01

NS, Nitrogen source; AL, Ammonia level.

^a−*d*^Means with different superscripts within a row differ (P < 0.05).

^1^Data were cubic root transformed to ensure normality of residuals. Values in parentheses are the means of untransformed data in individual treatments.

No interaction (*P* ≥ 0.18) of ammonia-N source with ammonia level was detected with respect to any of the bacterial phyla except for Actinobacteria (*P* < 0.01) and Fusobacteria (*P* = 0.01). There were significant differences in the relative abundance of Firmicutes, Proteobacteria, Fibrobacteres, Tenericutes, and Spirochaetes among different ammonia levels (*P* < 0.01). Compared with other ammonia treatment levels, the high TAN treatments (128 mmol/L) significantly increased Gram-positive Firmicutes and Actinobacteria (*P* < 0.05), while decreasing Gram-negative Fibrobacteres and Spirochaetes significantly (*P* < 0.05) and Gram-negative Bacteroidetes numerically (*P* > 0.05). These results indicate that some Gram-negative bacteria were sensitive, while Gram-positive bacteria were tolerant to high TAN treatment.

Different responses of rumen bacterial genera to the incremental additions of urea and NH_4_Cl are shown in Table 2. There was significant interaction (*P* ≤ 0.01) between nitrogen source and ammonia level with respect to the relative abundance of *Prevotellaceae* YAB2003 group, *Prevotella* 7, *Selenomonas, Eubacterium* eligens group, *Anaerovibrio, Sharpea, Escherichia-Shigella, Bifidobacterium*, and *Fusobacterium*, but not for others.

**Table 2:** Effects of NH_4_Cl and urea addition on the relative abundance of major ruminal bacterial genera (each with a relative abundance ≥1.0% in at least one treatment).

**Phylum**	**Genus/other**	**NH**_**4**_ **Cl**				**Urea**				**SEM**	***P*** **-value**		
		**A-0**	**A-8**	**A-32**	**A-128**	**U-0**	**U-8**	**U-32**	**U-128**		**NS**	**AL**	NS*AL
Bac	*Prevotella* 1	25.13	20.89	29.31	16.36	22.54	21.58	23.35	23.07	4.667	0.93	0.52	0.58
	*Rikenellaceae* RC9 gut group	4.10	4.45	4.05	2.25	5.25	5.26	4.52	2.85	0.685	0.13	< 0.01	0.96
	*Prevotellaceae* Ga6A1 group	3.95	2.93	4.72	1.27	3.65	3.23	3.04	0.81	0.716	0.30	< 0.01	0.58
	*Prevotellaceae* YAB2003 group	1.94^b^	1.77^b^	2.82^b^	4.13^a^	1.81^b^	1.75^b^	1.91^b^	1.31^b^	0.438	< 0.01	0.13	0.01
	*Bacteroidales* F082_norank	1.42	1.33	0.75	0.18	1.72	1.82	1.05	0.25	0.242	0.10	< 0.01	0.85
	*Muribaculaceae*_norank	1.23	1.00	0.80	1.85	1.14	1.02	0.77	1.83	0.219	0.85	< 0.01	0.99
	*Prevotellaceae* UCG-001	1.16	1.20	1.44	0.34	1.14	1.18	1.34	0.27	0.192	0.68	< 0.01	0.99
	*Prevotella* 7	0.51^b^	0.71^ab^	1.05^ab^	1.17^a^	0.45^b^	0.56^b^	0.37^b^	0.17^b^	0.129	< 0.01	0.32	< 0.01
Fir	*Agathobacter*	2.23	3.44	2.23	12.34	1.87	3.34	4.24	14.93	1.082	0.19	< 0.01	0.44
	*Anaerosporobacter*	2.13	1.28	0.46	1.89	2.57	1.89	0.87	1.95	0.329	0.11	< 0.01	0.86
	*Lachnospiraceae*_uncultured	1.98	1.94	0.90	0.88	1.67	1.81	1.99	2.15	0.434	0.13	0.69	0.18
	*Pseudobutyrivibrio*	1.92	1.75	1.80	3.58	1.99	2.06	1.72	2.25	0.361	0.33	0.01	0.13
	*Butyrivibrio* 2	1.04	0.73	0.72	0.97	1.00	0.83	0.68	1.45	0.188	0.34	0.045	0.49
	*Selenomonas*	0.80^b^	0.77^b^	1.06^b^	4.24^a^	0.70^b^	0.29^b^	1.11^b^	1.39^b^	0.258	< 0.01	< 0.01	< 0.01
	*Ruminococcus* 1	0.57	0.68	0.60	1.36	0.69	0.73	0.85	1.03	0.146	0.84	< 0.01	0.25
	*Ruminococcus* 2	0.52	0.80	0.65	0.92	0.91	1.09	0.88	1.42	0.213	0.03	0.16	0.93
	*Eubacterium* eligens group	0.49^c^	0.54^c^	0.46^c^	2.67^a^	0.41^c^	0.30^c^	0.49^c^	1.50^b^	0.154	< 0.01	< 0.01	< 0.01
	*Anaerovibrio*	0.47^b^	0.35^b^	0.33^b^	0.34^b^	0.51^b^	0.26^b^	0.38^b^	1.08^a^	0.076	< 0.01	< 0.01	< 0.01
	*Sharpea*	0.15^c^	0.25^c^	0.22^c^	2.81^a^	0.29^c^	0.31^c^	0.24^c^	1.31^b^	0.142	< 0.01	< 0.01	< 0.01
Pro	*Ruminobacter*	9.75	22.12	13.82	11.60	8.73	20.29	15.80	10.32	3.121	0.81	< 0.01	0.93
	*Succinivibrio*	1.77	2.14	1.78	1.99	1.96	1.53	1.95	2.27	0.279	0.99	0.69	0.37
	*Escherichia*−*Shigella*^1^	0.26^b^ (0.02)	0.27^b^ (0.02)	0.28^b^ (0.02)	0.38^b^ (0.06)	0.24^b^ (0.01)	0.25^b^ (0.02)	0.38^b^ (0.06)	1.06^a^ (1.25)	0.035	< 0.01	< 0.01	< 0.01
Fib	*Fibrobacter*	8.97	4.73	7.09	3.43	9.00	8.03	6.86	0.03	1.466	0.94	< 0.01	0.18
Spi	*Treponema* 2	6.94	4.77	7.33	0.67	6.84	4.30	6.30	0.66	1.169	0.43	< 0.01	0.80
Ten	*Anaeroplasma*	6.64	3.19	1.70	1.66	6.87	4.25	3.90	5.04	0.820	< 0.01	< 0.01	0.27
Act	*Bifidobacterium* ^1^	0.92^c^ (0.82)	0.98^c^ (0.94)	0.94^c^ (0.87)	2.00^a^ (8.11)	1.04^c^ (1.14)	0.90^c^ (0.79)	1.04^c^ (1.12)	1.60^b^ (4.29)	0.070	0.23	< 0.01	< 0.01
Fus	*Fusobacterium* ^1^	0.33^d^ (0.04)	0.46^cd^ (0.10)	0.60^c^ (0.23)	0.99^b^ (1.01)	0.35^d^ (0.04)	0.47^cd^ (0.12)	0.62^c^ (0.25)	1.32^a^ (2.42)	0.051	0.01	< 0.01	0.01

NS, Nitrogen source; AL, Ammonia level; Bac, Bacteroidetes; Fir, Firmicutes; Pro, Proteobacteria; Fib, Fibrobacteres; Spi, Spirochaetes; Ten, Tenericutes; Act, Actinobacteria; Fus, Fusobacteria.

^a−*d*^Means with different superscripts within a row differ (P < 0.05).

^1^Data were cubic root transformed to ensure normality of residuals. Values in parentheses are the means of untransformed data in individual treatments.

### Correlation analysis

**Figure 5:**
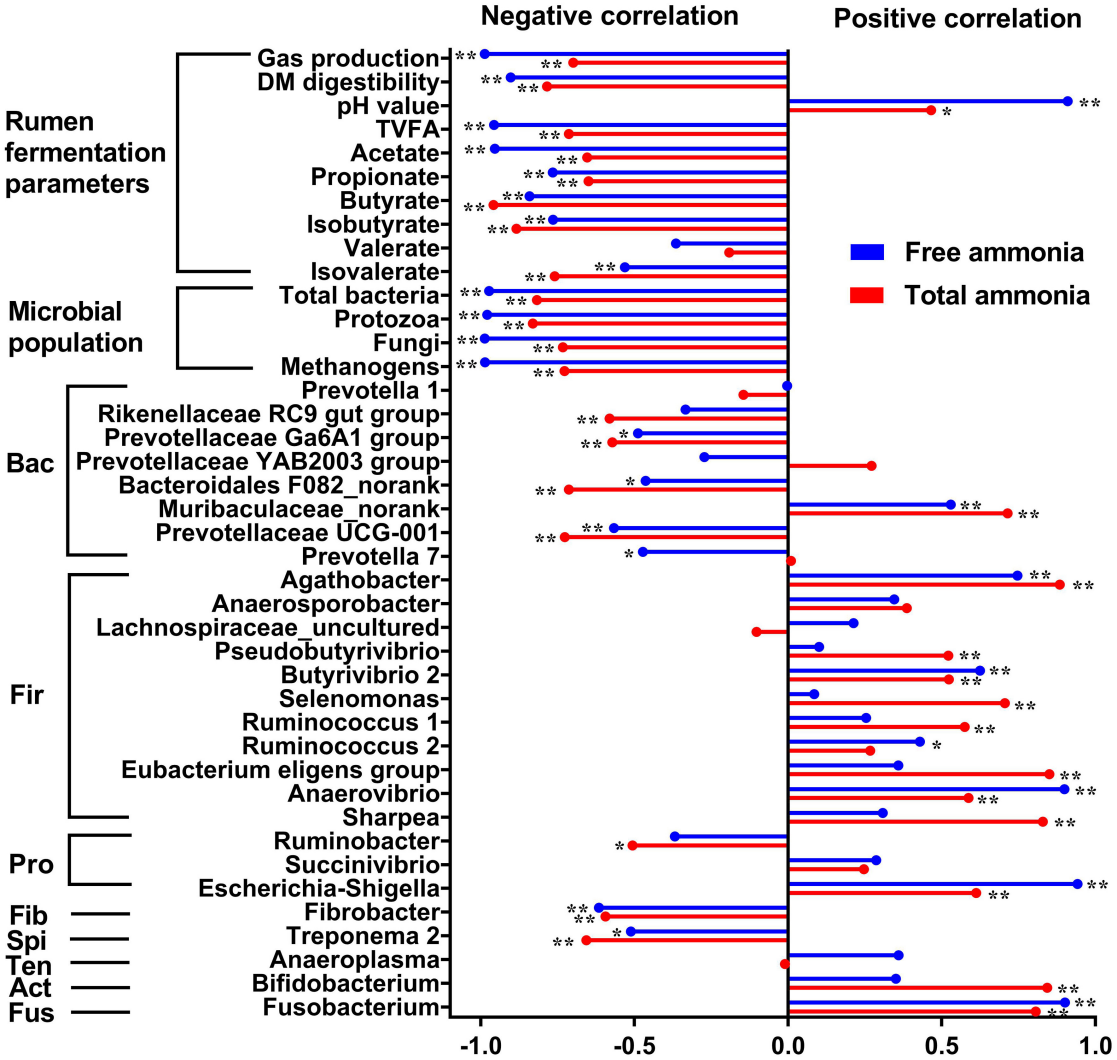
Pearson correlation coefficients (*r*) between total ammonia (red bars) or free ammonia (blue bars) and *in vitro* rumen fermentation parameters, absolute abundance of microbial populations (total bacteria, protozoa, fungi, and methanogens), the relative abundance of rumen bacteria at the genus level. *P < 0.05; **P < 0.01. Bac, Bacteroidetes; Fir, Firmicutes; Pro, Proteobacteria; Fib, Fibrobacteres; Spi, Spirochaetes; Ten, Tenericutes; Act, Actinobacteria; Fus, Fusobacteria.

To explore the correlations between TAN or FAN and *in vitro* rumen fermentation parameters, absolute abundance of microbial populations, the relative abundance of rumen bacteria at the genus level, Pearson's correlation analysis was performed. Pearson correlation analysis revealed a strong negative correlation between FAN and microbial populations quantified (i.e., total bacteria, protozoa, fungi, and methanogens) and *in vitro* rumen fermentation profiles (gas production, DM digestibility, total VFA, acetate, propionate, etc.) and a much weaker negative correlation between TAN and the above indicators (Figure 5). The correlation analysis between the relative abundance of bacterial genera and ammonia concentration (FAN and TAN, Figure 5) revealed the difference in tolerance to high ammonia between Gram-positive and Gram-negative bacteria. Most of the Gram-positive bacterial genera including *Agathobacter, Pseudobutyrivibrio, Butyrivibrio* 2, *Selenomonas, Ruminococcus* 1, *Ruminococcus* 2, *Eubacterium* eligens group, *Anaerobibrio, Sharpea*, and *Bifidobacterium* were positively correlated with FAN or TAN concentration (*P* < 0.05). In contrast, most of the Gram-negative bacterial genera including *Rikenellaceae* RC9 gut group, *Prevotellaceae* Ga6A1 group, *Bacteroidales* F082_norank, *Prevotellaceae* UCG-001, *Ruminobacter, Fibrobacter*, and *Treponema* were negatively correlated with the concentration of FAN or TAN concentration (*P* < 0.05). However, there are also a few exceptions. For example, Gram-negative bacteria of *Muribaculaceae*_norank, *Escherichia-Shigella*, and *Fusobacterium* also showed a positive correlation with FAN or TAN concentration (*P* < 0.05).

## Discussion

Ammonia and VFA are continuously produced by the rumen microbiota and then absorbed through the rumen wall. On the other hand, urea is constantly diffused back to the rumen. Therefore, an *in vitro* system was used to avoid the interference of absorption and diffusion across the rumen epithelia. The use of an *in vitro* system also allowed us to precisely control and test different levels of NH_4_Cl and urea. Previous studies have demonstrated that high FAN induced rapid absorption of ammonia through the rumen epithelium leading to ammonia toxicity (10, 16), but no studies had reported the effects of high FAN on rumen microbiota and fermentation. Combining high-throughput sequencing and real-time qPCR using an *in vitro* fermentation system, this study for the first time evaluated the effects of high FAN on the rumen microbiota and fermentation. Moreover, this study also provided a practical guidance for the utilization of NPN to improve rumen fermentation.

In this study, it was found that the time required for complete hydrolysis of different doses of urea varied. Helmer and Bartley (36) reviewed previous literature and reported that 100 mL or g of rumen liquid/content could convert 80–100 mg of urea to ammonia per h, which is a rate much higher than that observed in the present study with a similar addition dose (U-32, 96 mg/100 mL). This could be explained by the small amount of rumen fluid inoculated [buffer medium: rumen fluid inoculum ratio = 9:1 (v/v)]. The initial microbial population was much lower than that of the rumen content, thus taking longer to hydrolyze the high doses of urea. In addition, high ammonia may inhibit urease activity (37), thus reducing the rate of urea hydrolysis. In order to increase the *in vitro* urea hydrolysis rate, large inoculum can be used in future research.

In the present study, the increase of rumen pH after urea hydrolysis is consistent with our recent *in vitro* study (38). Previous *in vivo* studies also found that infusion of urea into the rumen of Jersey cows caused an elevated rumen pH (39). In contrast, in line with our *in vitro* study with NH_4_Cl addition, a previous ruminal NH_4_Cl infusion study in Holstein cows also found reduced rumen pH (17). Moreover, in a study on anaerobic digestion for biogas production, similar changes of pH after urea and NH_4_Cl addition were also found (18). The above results indicate that a pH and ammonia level model was successfully implemented by adding different doses of urea and NH_4_Cl. This model can help examine the effect of varying FAN levels on the rumen microbiome (both individual taxa and functional guilds such as fibrolytic bacteria) and on *in vitro* rumen fermentation.

Under normal rumen environmental condition, the rumen pH is typically below the pKa (9.21) of ammonia, and thus virtually all ammonia is present in the rumen as NH${}_{4}^{+}$ (40). However, as calculated according to Henderson-Hasselbalch equation (9), the amount of TAN present as NH_3_ varies almost exponentially as a function of pH (41). Thus, in the present study, the increased *in vitro* rumen pH in response to the urea addition resulted in a much higher FAN concentration compared to NH_4_Cl at a similar TAN level. Therefore, pH value is the key factor to determine the concentration of FAN in the rumen. For ruminants, the rumen pH is greatly influenced by dietary forage to concentrate ratio and buffers such as bicarbonate, calcium carbonate, and magnesium oxide (3). Thus, the rumen FAN level can be controlled to some extent by controlling the pH through modifying the buffer and diet composition.

Ammonia-N is an essential nutrient for microbial growth. The rumen microbiota needs 5–11 mmol/L ammonia to maximize microbial protein (42). Apparently, the reduced *in vitro* rumen fermentation in the groups of A-0 and U-0 was due to ammonia-N deficiency. High ammonia stress is a main issue in anaerobic digestion for biogas production (9). In a previous review, Jiang et al. (13) reported that the inhibitory concentrations of TAN for anaerobic digestion varied greatly, but if converted to FAN the inhibitory concentrations were more consistent. In livestock production, feeding a large amount of NPN to ruminants will create high ammonia stress to the animals, which increases the risk of ammonia toxicity (8). However, the effects of high FAN on *in vitro* rumen fermentation are unknown. In the present study, the FAN concentration of 0.31 mmol/L in the U-32 group resulted in some inhibition of *in vitro* rumen fermentation (gas production, DM digestibility, total VFA concentration, etc.), and the increased FAN concentration of group A-128 (0.57 mmol/L) and U-128 (3.96 mmol/L) increased the inhibition magnitude significantly. Thus, to ensure efficient rumen fermentation, the rumen FAN level should be controlled. Moreover, based on the above results, it is speculated that rumen FAN level could serve as a potential biological marker to monitor rumen fermentation, but more studies are needed to confirm the inhibition threshold.

Rumen microorganisms are solely responsible for feed degradation and VFA production (43), and rumen bacteria are the predominant contributors (44). In addition, rumen protozoa, fungi, and methanogens also play important but different roles in rumen digestion and metabolism (45). In the present study, the stronger negative correlation between FAN and microbial populations quantified (i.e., total bacteria, protozoa, fungi, and methanogens) and *in vitro* rumen fermentation profiles (gas production, DM digestibility, total VFA, acetate, propionate, etc.) and a much weaker correlation between TAN and the above indicators indicate that high FAN inhibited *in vitro* rumen fermentation by reducing microbial populations. However, the mechanism(s) by which high FAN inhibited rumen microbes remain to be elucidated. Reviews of anaerobic digestion for biogas production reported that at least two possible mechanisms were underpinning the inhibition of anaerobic digestion by high ammonia (13): (1) direct inhibition of some enzymes in the cytoplasm of microorganisms, and (2) alteration of the intracellular environment upon absorption of free ammonia, resulting in ammonia toxicity to microorganisms. The operation environment of anaerobic digesters for biogas production (pH, temperature, microbial composition, etc.) is quite different from that of the rumen. Therefore, further research efforts are needed to elucidate the mechanism(s) of ammonia inhibition to rumen microorganisms.

The performance of rumen fermentation is not only related to the total bacteria population but also closely related to the rumen bacterial community structure. Generally, *Ruminococcus albus, R. flavefaciens, Fibrobacter Succinogenes, Butyrivibrio fibrisolvens*, and *Eubacterium cellulosolvens* are considered the major cellulolytic bacterial species cultured (46). Besides, a forage incubation study indicated that some unclassified bacteria assigned to the families *Lachnospiraceae, Christensenellaceae, Ruminococcaceae, Rikenellaceae, Prevotellaceae*, and *Bacteroidales* mightly also play an important role in fiber degradation in the rumen (47). In the present study, the reduced abundance of potential fibrolytic bacterial genera (e.g., *Rikenellaceae* RC9 gut group, *Prevotellaceae* Ga6A1 group, *Bacteroidales* F082_norank, *Prevotellaceae* UCG-001, and *Fibrobacter*) might have partially explained the decreased DM digestibility in the high ammonia treatments. However, some potential fibrolytic bacterial genera such as *Pseudobutyrivibrio, Butyrivibrio* 2, and *Ruminococcus* 1 were increased by the high ammonia treatments. This may be attributed to the different tolerance of fibrolytic bacteria to high ammonia stress. However, it should be noted that an increase or decrease of the relative abundance of a bacterial genus does not necessarily mean an increase or decrease of its absolute abundance. Quantitative analysis of absolute abundance using qPCR or other quantitative methods can determine how these genera respond to high ammonia stress.

In the present study, the correlation results between the relative abundance of bacterial genera and ammonia concentration indicate that some Gram-negative bacteria were sensitive, while Gram-positive bacteria were tolerant to high TAN treatment. However, to the best of the authors' knowledge, no literature to date has reported the tolerance response of Gram-positive vs. Gram-negative bacteria to high ammonia stress. Therefore, the difference in tolerance to high ammonia stress between Gram-positive and Gram-negative bacteria warrants future research.

## Conclusion

In the present study, an *in vitro* rumen pH and ammonia difference model was implemented by adding urea and NH_4_Cl. Urea hydrolysis increased, while NH_4_Cl dissociation slightly reduced *in vitro* rumen pH. At the same TAN level, the increased rumen pH by urea addition resulted in much higher FAN concentrations compared to NH_4_Cl addition. High FAN inhibited *in vitro* rumen fermentation by reducing the absolute abundance of total bacteria, fungi, protozoa, and methanogens. Additionally, bacterial community structure changed differently in response to nitrogen source and ammonia level. This study demonstrated that the inhibition of high ammonia to *in vitro* rumen fermentation is pH dependent.

## Data availability statement

The datasets presented in this study can be found in online repositories. The names of the repository/repositories and accession number(s) can be found at: https://www.ncbi.nlm.nih.gov/, PRJNA940661.

## Ethics statement

The animal study was reviewed and approved by Animal Care and Use Committee of Nanjing Agricultural University (protocol number: SYXK2017-0007). Written informed consent was obtained from the owners for the participation of their animals in this study.

## Author contributions

JS conceived and designed the experiments and wrote the paper. WZ and YX performed the experiments. JS, WZ, and YX analyzed the data. ZY revised the paper. All authors contributed to the article and approved the submitted version.
