# Supplementary material for: The inhibition of high ammonia to in vitro rumen fermentation is pH dependent
Source: Front Vet Sci. 2023 Mar 30;10:1163021. doi: 10.3389/fvets.2023.1163021 (PMC10097989; doi:10.3389/fvets.2023.1163021)
Supplement: Supplementary file 1 [file Table_1.DOCX]

**Supplementary files**

Table S1. Primers used for real-time PCR quantification of rumen target organisms

| Target organisms | Primers sequences (5'→3') | Annealing temp. (°C) | Amplicon length (bp) | References |
| --- | --- | --- | --- | --- |
| Total bacteria | F: CGGCAACGAGCGCAACCC | 60 | 161 | Denman and McSweeney (2006) |
|  | R: CCATTGTAGCACGTGTGTAGCC |  |  |  |
| Fungi | F: GAGGAAGTAAAAGTCGTAACAAGGTTTC | 60 | 120 | Denman and McSweeney (2006) |
|  | R: CAAATTCACAAAGGGTAGGATGATT |  |  |  |
| Protozoa | F: GCTTTCCGWTGGTAGTGTATT | 54 | 223 | Sylvester et al. (2004) |
|  | R: CTTGCCCTCYAATCGTWCT |  |  |  |
| Methanogens  (*mcr*A gene) | F: TTCGGTGGATCDCARAGRGC | 60 | 190 | Denman et al. (2007) |
|  | R: GBARGTCGWAWCCGTAGAATCC |  |  |  |
